# Typhoid Fever: Way Forward

**DOI:** 10.4269/ajtmh.18-0111

**Published:** 2018-07-25

**Authors:** Zulfiqar A. Bhutta, Michelle F. Gaffey, John A. Crump, Duncan Steele, Robert F. Breiman, Eric D. Mintz, Robert E. Black, Stephen P. Luby, Myron M. Levine

**Affiliations:** 1 Centre for Global Child Health, The Hospital for Sick Children, Toronto, Canada;; 2 Dalla Lana School of Public Health, University of Toronto, Toronto, Canada;; 3 Center of Excellence in Women and Child Health, The Aga Khan University, Karachi, Pakistan;; 4 Centre for International Health, University of Otago, Dunedin, New Zealand;; 5 Enteric and Diarrheal Diseases, Bill & Melinda Gates Foundation, Seattle, Washington;; 6 Emory Global Health Institute, Emory University, Atlanta, Georgia;; 7 National Center for Emerging and Zoonotic Infectious Diseases, Centers for Disease Control and Prevention, Atlanta, Georgia;; 8 Johns Hopkins Bloomberg School of Public Health, Baltimore, Maryland;; 9 Centre for Innovation in Global Health, Woods Institute for the Environment, Stanford University, Stanford, California;; 10 Global Health, Vaccinology and Infectious Diseases, University of Maryland School of Medicine, Baltimore, Maryland

## Abstract

The Tackling Typhoid supplement shows that typhoid fever continues to be a problem globally despite socioeconomic gains in certain settings. Morbidity remains high in many endemic countries, notably in sub-Saharan Africa and South Asia. In addition, antimicrobial resistance is a growing issue that poses a challenge for clinical management. The findings from this supplement revealed that outside of high-income countries, there were few reliable population-based estimates of typhoid and paratyphoid fever derived from surveillance systems. This indicates the need for monitoring systems that can also characterize the effectiveness of interventions, particularly in low- and middle-income settings. The country case studies indicated that gains in economic conditions, education, and environmental health may be associated with reductions in typhoid fever burden. Over the study period, the effect is mainly notable in countries with higher baseline levels of economic development, female literacy, and investments in public sanitation. High burden countries must continue to invest in strategies at the local level to address environmental factors such as access to safe drinking water and improved public sanitation that are known to interrupt transmission or diminish the risk of acquiring typhoid. Developing more effective vaccines and incorporating appropriate immunization strategies that target populations with the greatest risk could potentially alleviate disease burden.

## INTRODUCTION

As several articles in this series have shown, despite much historical progress globally, typhoid fever remains a major problem in several parts of the world. Although the burden has declined in many countries, the disease is still widespread, notably in South Asia and sub-Saharan Africa, and a source of much morbidity,^1^ loss in income and economic productivity,^2^ and, in many instances, severe disease requiring hospitalization. Although accurate estimates are difficult to come by, 200,000 global deaths may be associated with typhoid, predominantly in certain impoverished settings where incidence can be as high as one in five children experiencing typhoid fever by the age of 10.^3,4^ In recent years, the emergence of drug resistance, especially multidrug resistant– and fluoroquinolone- resistant strains of *Salmonella* Typhi (*S*. Typhi) and *Salmonella* Paratyphi A (*S*. Paratyphi A), has been shown to be associated with more severe disease and potentially adverse outcomes, posing challenges for clinical management and further increasing disease burden.^5^ Control measures should include investments in water and sanitation services, food safety, and optimal immunization strategies that countries can implement.^6^ Despite some specific global recommendations on the use of currently available typhoid vaccines in school age children, there are very few examples of systematic implementation; furthermore, currently available vaccines do not address the burden of *Salmonella* Paratyphi A disease. Similarly, attempts to expand access to potable water and to improve sanitation in many locales have been unable to keep pace with population growth and migration.

## MATERIALS AND METHODS

As the introductory article in this series describes,^6^ we undertook a global systematic review of typhoid fever incidence, morbidity, and mortality trends^7^ and conducted eight country case studies^8–15^ to evaluate country-level data from a range of contexts and regions. Relatively few low- and middle-income countries (LMICs) had blood culture–based national surveillance and reporting systems in place. Many relied on clinical case definitions, often in combination with diagnosis based on Widal serological testing.^16,17^ In areas where typhoid and paratyphoid A fever are both endemic, clinical case counts do not allow disease burdens to be ascribed to specific etiologic agents.

In contrast to other reports, we sought datasets with confirmed *Salmonella* Typhi bloodstream infections. We developed disease rate estimates using population or blood culture denominators for comparison and estimation of trends. The laboratory facilities or centers selected were generally representative of national facilities with diverse catchment populations and had quality assurance protocols in place.

Notwithstanding the limitation of scarce blood culture–based nationally representative surveillance systems in LMICs, we identified data from some countries with reliable national information systems that documented substantial reductions in typhoid occurrence over recent decades. These countries included Vietnam,^15^ Thailand,^14^ Chile,^9^ and South Africa.^13^ In other settings where typhoid fever trends were more uncertain, such as Pakistan,^12^ India,^10^ Bangladesh,^8^ and Nigeria,^11^ we obtained time trend information from large reference laboratories with standardized methods and documented denominators and relevant test volumes over time.

In an effort to understand the role of established determinants and risk factors for typhoid fever from various regions, we sought data on such factors within the country case studies. We also undertook standardized qualitative studies. These targeted qualitative studies with relevant stakeholders, were undertaken to understand some of the policy-relevant decisions and drivers of change.

## RESULTS: TREND ANALYSIS

Our findings revealed, as expected, that outside of high-income countries, there were very few reliable population-based estimates of typhoid fever derived from robust surveillance systems. Hence, regional estimates of typhoid fever burden and trends from many large geographic regions representing LMICs were not available. However, we found reasonably representative population estimates from Thailand, Chile, and South Africa (Table 1). Elsewhere, reliable data on laboratory-confirmed cases of typhoid and paratyphoid fever with time trends were only available from a few centers or laboratories (Tables 2 and 3). Consequently, time trend assessments are contingent on catchment populations and laboratory clientele.

**Table 1:** National surveillance data for typhoid fever

Country	2000–2004			2005–2009			2010–2015			2000–2015		
	Incidence per 100,000 pop.	AARR (%)	AAAR (per 100,000 pop.)	Incidence per 100,000 pop.	AARR (%)	AAAR (per 100,000 pop.)	Incidence per 100,000 pop.	AARR (%)	AAAR (per 100,000 pop.)	Incidence per 100,000 pop.	AARR (%)	AAAR (per 100,000 pop.)
Chile	2000: 5.57	−15.93	−0.583	2005: 2.93	−28.97	−0.427	2010: 1.16	−29.31	−0.17	2000: 5.57	−15.98	−0.404
	2004: 3.31			2009: 1.05			2012: 0.82			2012: 0.82		
South Africa	N/A	N/A	N/A	2005: 0.39	−30.66	−0.061	2010: 0.15	10.06	0.008	2004: 0.14	4.04	−0.011
	N/A	N/A	N/A	2009: 0.13			2014: 0.2			2014: 0.20		
Thailand	N/A	N/A	N/A	2005: 6.24	−2.52	−0.064	2010: 6.08	−20.63	−0.666	2003: 8.65	−9.93	−0.354
	N/A	N/A	N/A	2009: 5.78			2014: 3.04			2014: 3.04		

AAAR = average annual absolute reduction; AARR = average annual relative reduction; N/A = not applicable.

**Table 2:** Subnational data from institutions for typhoid

	2000–2004			2005–2009			2010–2015			2000–2015		
Country: institution	% Typhoid positive [*N*]	AARR (%)	AAAR (%)	% Typhoid positive [*N*]	AARR (%)	AAAR (%)	% Typhoid positive [*N*]	AARR (%)	AAAR (%)	% Typhoid positive [*N*]	AARR (%)	AAAR (%)
Bangladesh: Dhaka Shishu, Dhaka	2001: 0.91 [2,312]	94.7	0.734	2005: 4.3 [3,375]	−0.94	−0.08	2010: 4.31 [5,340]	−17.5	−0.508	2001: 0.91 [2,312]	8.49	0.108
	2004: 3.45 [3,105]			2009:4.18 [5,287]			2014: 2.42 [7,198]			2014: 2.42 [7,198]		
Bangladesh: Shishu Shasthya, Dhaka	N/A	N/A	N/A	N/A	N/A	N/A	2010: 3.06 [1.603]	−15.29	−0.084	2010: 3.06 [1,603]	−15.29	−0.084
	N/A	N/A	N/A	N/A	N/A	N/A	2014: 1.86 [2,095]			2014: 1.86 [2,095]		
Bangladesh:Popular diagnostics, Dhaka	2002: 3.93 [4,711]	123.4 1	2.425	2005: 4.02 [6,171]	24.3	0.442	2010: 6.78 [3,614]	−4.74	−0.275	2002: 3.93 [4,711]	3.7	0.009
	2004: 8.78 [4,739]			2009: 7.72 [3,225]			2014: 5.86 [3,565]			2014: 5.86 [3,565]		
India: all India Institute of Medical Sciences, New Delhi	2000: 0.63 [16,437]	−14.77	−0.074	2005: 0.32 [25,129]	−11.74	−0.024	2010: 0.43 [23,403]	−14.48	−0.0454	2000: 0.63 [16,437]	−6.94	−0.032
	2004: 0.39 [25,473]			2009: 0.22 [25,603]			2015: 0.23 [13,474]			2015: 0.23 [13,474]		
India: Christian Medical College, Vellore	2000: 1.39 [13,204]	−34.53	−0.309	2005: 0.48 [17,461]	−2.13	0.001	2010: 0.52 [33,897]	−31.11	−0.085	2000: 1.39 [13,204]	−14.92	−0.095
	2004: 0.39 [16,886]			2009: 0.45 [31,487]			2014: 0.17 [47,766]			2014: 0.17 [47,766]		
India: Nair Hospital, Mumbai	N/A	N/A	N/A	N/A	N/A	N/A	2010: 0.4 [3,265]	−13.38	−0.072	2009: 0.12 [2,575]	21.32	−0.012
	N/A	N/A	N/A	N/A	N/A	N/A	2014: 0.26 [3,057]			2014: 0.26 [3,057]		
Nigeria: Lagos State University Teaching hospital and general hospital Lagos	2000: 13.8 [130]	−14.25	−1.38	2005: 11 [254]	2.02	−0.39	2010: 10.6 [169]	6.25	−0.3	2000: 13.8 [130]	1.58	−0.18
	2004: 8.7 [299]			2009: 11.7 [213]			2015: 9.6 [115]			2015: 9.6 [115]		
Nigeria: Aminu Kano Teaching hospital, Hasiya Bayero Pediatric hospital, and Murtala Specialist hospital	N/A	N/A	N/A	N/A	N/A	N/A	2013: 3.9 [1,274]	164	3.2	2013: 3.9 [1,274]	31.1	1.38
	N/A	N/A	N/A	N/A	N/A	N/A	2015: 10.3 [4,080]			2016: 6.7 [3,840]		
Nigeria: NHA, UATHG, NDH, ZMC;GHA, MHA, FMCK	N/A	N/A	N/A	N/A	N/A	N/A	2010: 5.1 [723]	−37.1	−0.57	2008: 1.2 [85]	1.15	−0.16
	N/A	N/A	N/A	N/A	N/A	N/A	2015: 0.8 [2,326]			2016: 1.3 [2,014]		
Pakistan: Armed Forces Institute of Pathology, Rawalpindi	N/A	N/A	N/A	2005: 0.73 [2,317]	24.54	0.138	2010: 1.12 [2,062]	−8.94	−0.0323	2004: 0.80 [2,859]	−0.38	−0.014
	N/A	N/A	N/A	2009: 1.41 [2,341]			2015: 0.77 [4,161]			2015: 0.77 [4,161]		
Pakistan: The Aga Khan University, Karachi	2000: 1.76 [18,266]	3.83	−0.124	2005: 1.62 [34,915]	17.68	0.26	2010: 1.86 [39,070]	−2.22	−0.077	2000: 1.76 [18,266]	−0.25	−0.053
	2004: 1.97 [31,020]			2009: 2.64 [37,828]			2015: 1.7 [37,635]			2015: 1.70 [37,635]		
Pakistan: Shaukat Khanum Memorial Cancer hospital, Lahore	N/A	N/A	N/A	2005: 0.53 [5,494]	−7.46	0.008	2010: 0.69 [8,081]	−17.94	−0.054	2005: 0.53 [5,494]	−3.76	−0.005
	N/A	N/A	N/A	2009: 0.42 [7,910]			2014: 0.39 [16,001]			2014: 0.39 [16,001]		
Pakistan: The Aga Khan University, Hyderabad	2000: 4 [302]	−19.3	−0.25	2005: 2.2 [415]	−10.1	−0.28	2010: 2.5 [1,297]	−3.15	−0.063	2000: 4 [302]	−4.18	−0.092
	2004: 2.1 [424]			2009: 1.6 [867]			2015: 2.2 [3,135]			2015: 2.2 [3,135]		
Vietnam: hospital for Tropical diseases, Ho Chi Minh city	2000: 2.67 [3,596]	−24.7	−0.415	2005: 0.63 [4,764]	−20.21	−0.017	2010: 0.24 [8,777]	−33.86	−0.041	2000: 2.67 [3,596]	−24.43	−0.176
	2004: 1.14 [4,554]			2009: 0.32 [7,543]			2014: 0.07 [9,631]			2014: 0.07 [9,631]		

AAAR = average annual absolute reduction; AARR = average annual relative reduction; FMCK = Federal Medical Center Keffi; GHA = Garki Hospital Abuja; [*N*] refers to the sample size that is, the total number of specimens analyzed in the given year; MHA = Maitama Hospital Abuja; NDH = Nyanya District Hospital; NHA = The National Hospital, Abuja; UATHG = University of Abuja Teaching Hospital, Gwagwalada; ZMC = Zankli Medical Center.

**Table 3:** National surveillance and institutional data for paratyphoid fever

Country: institution	2000–2004			2005–2009			2010–2015			2000–2015		
	Incidence per 100,000 pop.	AARR (%)	AAAR (per 100, 000 pop)	Incidence per 100,000 pop.	AARR (%)	AAAR (per 100, 000 pop)	Incidence per 100,000 pop.	AARR (%)	AAAR (per 100, 000 pop)	Incidence per 100,000 pop.	AARR (%)	AAAR (per 100, 000 pop)
Thailand*	N/A	N/A	N/A	2005:0.4 4	20.51	0.062	2010: 0.59	−7.96%	−0.034	2003: 0.55	−1.77%	−0.0002
	N/A	N/A	N/A	2009: 0.77			2014: 0.46			2014: 0.46		
	% Paratyphoid Positive [*N*]	AARR (%)	AAAR (%)	% Paratyphoid Positive [*N*]	AARR (%)	AAAR (%)	% Paratyphoid Positive [*N*]	AARR (%)	AAAR (%)	% Paratyphoid Positive [*N*]	AARR (%)	AAAR (%)
Bangladesh: Dhaka Shishu, Dhaka	2001: 0.17 [2,312]	57.1 8	0.051	2005: 0.5 [3,375]	12.92	0.069	2010: 0.73 [5,340]	−27.34	−0.16	2001: 0.17 [2,312]	4.25	0.0317
	2004: 0.42 [3,105]			2009: 0.72 [5,287]			2014: 0.28 [7,198]			2014: 0.28 [7,198]		
Bangladesh: Shishu Shasthya, Dhaka	N/A	N/A	N/A	N/A	N/A	N/A	2010: 0.50	−1.35	−0.012	2010: 0.50 [1,603]	−1.35	−0.012
	N/A	N/A	N/A	N/A	N/A	N/A	2014: 0.48			2014: 0.48 [2,095]		
Bangladesh: Popular diagnostics, Dhaka	2002:1.55 [4,711]	87.7 4	0.68	2005: 0.76 [6,171]	36.9	0.245	2010: 2.99 [3,614]	−12.69	−0.336	2002: 1.55 [4,711]	2.3	0.0777
	2004: 2.91 [4,739]			2009: 1.95 [3,225]			2014: 1.99 [3,565]			2014: 1.99 [3,565]		
India: all India Institute of Medical Sciences, New Delhi	2000: 0.13 [16,437]	9.35	0.013	2005: 0.11 [25,129]	−18.29	−0.017	2010: 0.05 [23,403]	35.79	0.0166	2000: 0.13 [16,437]	1.93	−0.0087
	2004: 0.17 [25,473]			2009: 0.06 [25,603]			2015: 0.17 [13,474]			2015: 0.17 [13,474]		
India: Nair hospital, Mumbai	N/A	N/A	N/A	N/A	N/A	N/A	2010: 0.03 [3,265]	32.64	0.0097	2009: 0.04 [2,575]	165.58	0.0097
	N/A	N/A	N/A	N/A	N/A	N/A	2014: 0.07 [3,057]			2014: 1.99 [3,057]		
Pakistan: The Aga Khan University, Karachi	2000: 0.8 [18,266]	2.44	−0.075	2005: 0.59 [34,915]	46.63	0.339	2010: 1.09 [39,070]	−17.7	−0.168	2000: 0.80 [18,266]	−3.3	−0.0101
	2004:0.86 [31,020]			2009: 1.86 [37,828]			2015: 0.5 [37,635]			2015: 0.50 [37,635]		
Pakistan: The Aga Khan University, Hyderabad	2000: 1.66 [302]	−0.2	−0.078	2005: 0.48 [415]	6.51	0.27	2010: 0.39 [1,297]	−0.65	−0.007	2000: 1.66 [302]	−10	−0.044
	2004: 1.65 [424]			2009: 0.58 [867]			2015: 0.38 [3,135]			2015: 0.38 [3,135]		

AAAR = average annual absolute reduction; AARR = average annual relative reduction; [*N*] refers to the sample size that is, the total number of specimens analyzed in the given year.

* National.

Data from longstanding collection systems in Thailand, and those from a national surveillance system in Chile, show a clear reduction in typhoid fever occurrence over time, with annual rates of relative reduction approximating 10% and 16%, respectively. The average annual absolute reduction was also comparable in these countries with the incidence of typhoid fever falling by approximately 0.4 cases per 100,000 every year between 2000 and 2014 (Table 1). By contrast, the national enteric diseases surveillance system in South Africa showed no overall reduction in typhoid fever incidence between 2003 and 2014, and instead showed a relative increase of 4%. The latter summative figure did not capture wide variations between regions over time or reductions that might have occurred in time periods before robust national surveillance was in place.

**Figure 1.**
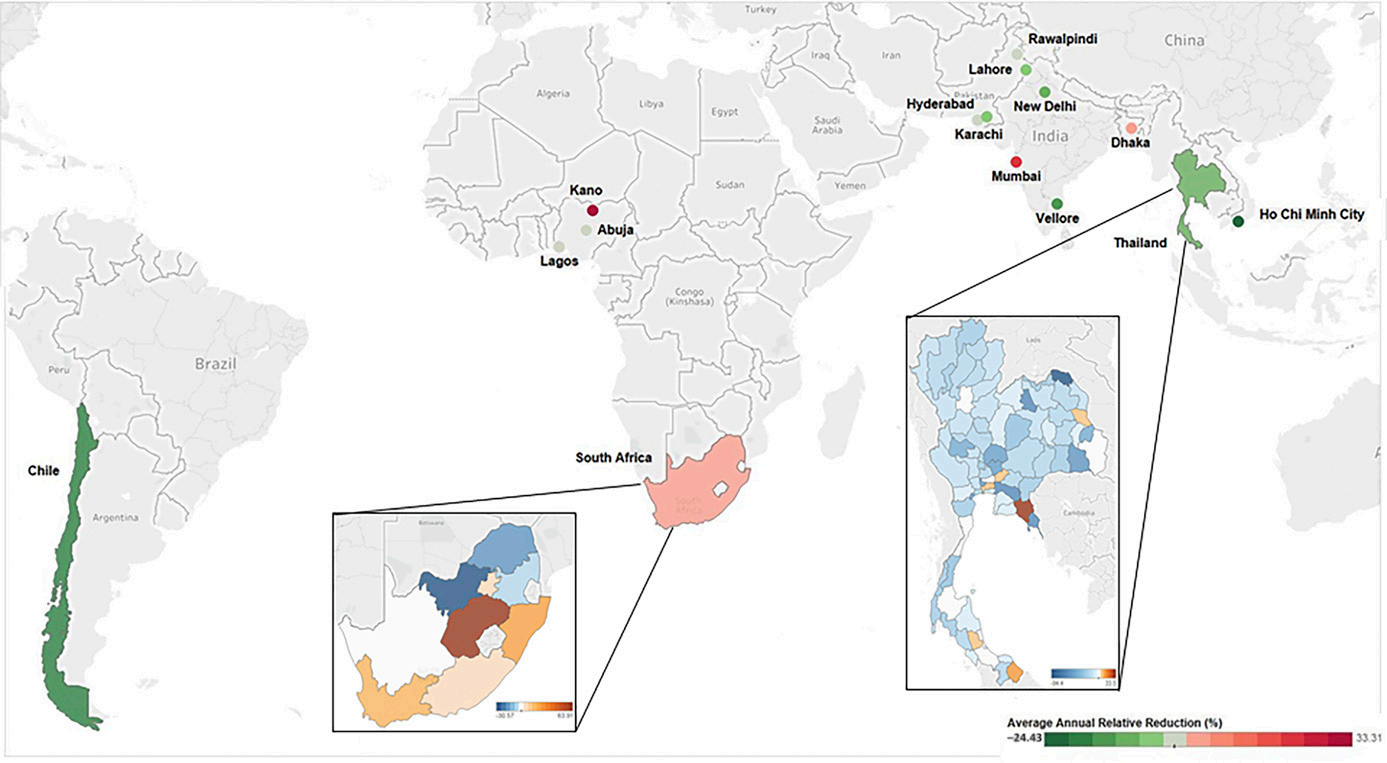
Average annual rate of reduction across eight case countries with subnational estimates. This figure shows the percentage for the average annual relative reduction in typhoid in Bangladesh, Chile, India, Nigeria, Pakistan, and Vietnam. The color gradient ranges from −24.43, shown in dark green, indicating reduction in typhoid to 33.31, shown in dark red, indicating an increase in typhoid incidence. In South Africa the gradient ranges from −17.07 (dark blue) to 63.91 (brown) sub-nationally. In Thailand the gradient ranges from −94.39 (dark blue) to 31.63 (brown) sub-nationally.

The country studies with sub-national data also present interesting and, in some instances, contrasting findings. The largest reduction in the isolation of *Salmonella* Typhi from blood cultures came from the Hospital for Tropical Diseases in Ho Chi Minh City, Vietnam, where the average annual relative reduction was 24.4% (Figure 1). Beginning in 2000, the percentage of *Salmonella* Typhi isolated from blood cultures decreased by approximately 0.18% from year to year. In Nigeria, a serial evaluation of blood culture–confirmed typhoid fever from two major institutions in Lagos over a 15-year period showed an increase in the proportion of blood cultures positive for *Salmonella* Typhi of approximately 0.7% from 1 year to the next. By contrast, in Pakistan, serial data from three major laboratory systems from Karachi in the south, Lahore in Punjab, and the Central Armed Forces laboratories in Rawalpindi show consistent trends in reduction of the relative proportion of blood cultures positive for *Salmonella* Typhi over time. At The Aga Khan University Hospital laboratory services, which has maintained prospective records on *Salmonella* Typhi isolation for over two decades, the annual reduction in the proportion of blood cultures positive for *Salmonella* Typhi averaged 0.25% annually from 2000 to 2015. A comparable reduction in isolation was documented at the All India Institute of Medical Sciences in Delhi and the Christian Medical College laboratories in Vellore, suggesting that academic centers with stable laboratory systems and catchment populations might provide a reasonable spotlight on trends over time. These trends were by no means universal for South Asia. In Dhaka, Bangladesh, there was an increase in the proportion of *Salmonella* Typhi–positive isolates over time at the two major laboratories in Bangladesh serving the Dhaka Shishu Hospital and the Popular Diagnostics Laboratories with an average annual increase of approximately 9% from Dhaka Shishu and 4% from Popular Diagnostics. The same was notable for the BYR Nair Charitable Hospital in Mumbai, where the proportion of blood cultures positive for *Salmonella* Typhi displayed an average relative increase of approximately 21% from 2009 to 2014.

### Determinants.

Although direct association is difficult to prove, our review shows that despite wide variations, typhoid fever has declined dramatically in countries with rapid rises in socioeconomic status, such as Vietnam, Thailand, and Chile (Table 4). However, these changes were associated with two key factors in Chile^9^ at outset, namely, high rates of female literacy ranging 94–96% from 1990 to 2011 and relatively high levels of improved sanitation at coverage ranging 72–86% from 1990 to 2012. It must be recognized that in Santiago, this was accomplished by additional massive wastewater treatment plants that eliminated the use of raw sewage as fertilizer on local crops. Although relatively high female literacy rates (> 70%) in Thailand and Vietnam along with relatively high levels of improved sanitation coverage in Thailand (> 70%) were also observed, the association was difficult to discern.^14,15^ By contrast, despite comparable or higher economic gains, India, Pakistan, and Bangladesh, as well as Nigeria, have a persistently high typhoid problem, suggesting that the benefits of economic gains were not effective in reducing exposure to *Salmonella* Typhi or the clinical consequences thereof. In all of these countries, there are continuing disparities and unequal access to improved water and sanitation facilities. We did not observe a clear association with access to improved water sources in any country. However, it is widely understood that the current indicator of “improved water” as used by the Joint Monitoring Program of World Health Organization and United Nations Children’s Fund does not accurately distinguish microbiologically safe water from water that is contaminated with human feces,^18,19^ nor does it take into account important issues such as intermittency of supply. Although often difficult to measure, the Typhoid Surveillance in Africa Programme supplement published in 2016 outlines the importance of unsafe water as a risk factor for typhoid fever transmission in Africa.^20^ Similarly, current information on access to improved sanitation is largely related to access to toilets as opposed to the entire range of sanitation and sewage disposal infrastructure. Provision of clean and safe toilets is an important step, however, as in South Asia, substantial proportions of the population, averaging 25–40% over the last decade, practice open defecation given the lack of facilities (Table 4). These poor conditions associated with lack of sanitation have been shown to be associated with high rates of stunting.^21,22^ Similarly high rates of open defecation are found in Nigeria and many other countries in sub-Saharan Africa.^23^

**Table 4:** Summary of contextual factor trends in eight case countries

Country	Start and end year	Contextual factor									
		Poverty rate (% population living on < $1.90/day)	Average annual rate of change	Adult female literacy rate	Average annual rate of change	Access to improved sanitation	Average annual rate of change	Access to improved water supply	Average annual rate of change	Diarrheal mortality in children < 5 (rate per 100 live births)	Average annual rate of change
Bangladesh	Year 1	1991: 72%	−2.8	1991: 26%	+3.78	1990: 34%	+2.47	1990: 68%	+1.03	2000: 11.11	−11.34
	Year 2	2010: 44%	–	2013: 56%	–	2015: 61%	–	2015: 87%	–	2013: 2.62	–
Chile*	Year 1	1990: 13%	−7.4	1990: 94%	+0.11	1990: 72%	+0.93	–	–	2000: 0.1	−12.55
	Year 2	2011: 3%	–	2011: 96%	–	2012: 87%	–	–	–	2013: 0.02	–
India	Year 1	1993: 46%	−4.4	1991: 34%	+2.97	1990: 17%	+3.64	1990: 71%	+1.21	2000: 13.38	−7.15
	Year 2	2011: 21%	–	2006: 51%	–	2015: 40%	–	2015: 94%	–	2013: 5.49	–
Nigeria	Year 1	1992: 57%	−0.4	1991: 44%	+0.56	1990: 38%	−1.12	1990: 40%	+2.30	2000: 22.76	−5.88
	Year 2	2009: 53%	–	2015: 50%	–	2015: 29%	–	2015: 69%	–	2013: 11	–
Pakistan	Year 1	1990: 59%	−9.8	1998: 29%	+3.08	1990: 24%	+4.17	1990: 86%	+0.24	2000: 16.86	−5.12
	Year 2	2010: 8.3%	–	2012: 43%	–	2015: 64%	–	2015: 91%	–	2013: 8.97	–
South Africa	Year 1	1993: 32%	−3.8	1996: 81%	+0.90	1990: 51%	+1.05	1990: 83%	+0.48	2000: 7.19	−6.45
	Year 2	2011: 17%	–	2012: 93%	–	2015: 66%	–	2015: 93%	–	2013: 3.23	–
Thailand	Year 1	1990: 9%	−21.4	2000: 91%	+0.47	1990: 87%	+0.28	1990: 87%	+0.47	2000: 1.28	−10.03
	Year 2	2012: 0.06%	–	2015: 97%	–	2015: 93%	–	2015: 98%	–	2013: 0.36	–
Vietnam	Year 1	1992: 49%	−13.4	1999: 87%	+0.56	1990: 36%	+3.27	1990: 63%	+1.84	2000: 5.52	−5.28
	Year 2	2012: 3%	–	2009: 91%	–	2015: 78%	–	2015: 98%	–	2013: 2.88	–

* Chile reports poverty as percent poor and literacy is not gender stratified. Estimates for poverty rate, literacy rate, and access to improved sanitation were obtained from the Ministry of Health and National Statistics Institute 1) WorldBank, 2) WHO/UNICEF Joint Monitoring Program, 3) United Nations Educational, Scientific and Cultural Organization (UNESCO); 4) WorldBank Global Poverty Working Group, 5) Global, regional, and national causes of child mortality in 2000–2013, with projections to inform post-2015 priorities: an updated systematic analysis.

#### Health sector and food safety interventions.

The relationship between typhoid fever trends and health sector interventions in the eight case countries is not very clear. Vaccination strategies were implemented or evaluated in several of the countries we evaluated, including Thailand, Vietnam, and, Chile but generally in targeted geographic subpopulations. In the Metropolitan Region of Chile (i.e., Santiago), large-scale field trials, including an effectiveness trial, were carried out in several administrative areas of the city during the 1980s with the live oral typhoid vaccine Ty21a, and clear reductions of typhoid fever in the school-age population of those areas were observed. An abrupt and precipitous reduction of typhoid fever in Santiago was observed beginning in 1991 following a governmental prohibition of the use of raw sewage water to irrigate crops (mainly vegetables consumed uncooked in salads) that were grown on farms in the Metropolitan Region. Environmental bacteriological studies had indicated that this practice of irrigation with raw sewage during the summer months was responsible for the long-cycle transmission in the Metropolitan Region and maintained hyper-endemicity of typhoid.^24^ This strict prohibition was instituted following an outbreak of El Tor cholera in Santiago in April 1991. The ban on irrigation of crops with untreated sewage water was accompanied by health education messages and enhancement of food safety inspections, and sanitation campaigns in the wake of the cholera outbreak.^9^ Clear temporal links of food safety measures with typhoid incidence changes in Thailand and Vietnam are less evident. In many South Asian countries, with sufficient evidence of the link between endemic typhoid or paratyphoid fever with poor hygiene and consumption of street foods,^25–29^ we found no evidence that sustained systematic interventions to screen for chronic carriers among food handlers were undertaken in any setting. In Pakistan, a study of a sample of food handlers in Karachi identified 9% as convalescent or chronic carriers.^30^ A study in India using nested polymerase chain reaction on cadaver gallbladder specimens identified 6–13% samples positive among males and females,^31^ whereas Vi serology identified 4–15% as potential carriers.^31^ None of the target countries with either low occurrence or persistent disease had any systematic programs for identifying chronic carriers or screening food handlers in place in the last 25 years, so their potential contribution to typhoid burden reduction could not be assessed.

The role of the school-based typhoid vaccination campaign in Thailand is more nuanced. The vaccination campaign was initiated in 1977, well before the occurrence of typhoid fever began to drop, with whole-cell typhoid vaccine in primary school children aged 5–12 years.^32^ However, a recent analysis of whole-genome sequencing of *Salmonella* Typhi isolates from the pre-vaccination period and the period thereafter suggested that most genotypes observed before the immunization program were not observed thereafter, and that subsequent isolates were likely related primarily to introductions by travelers from other countries in the region.^33^

The role of other health system interventions such as diagnostics and antimicrobial use is uncertain. None of the country experts interviewed as part of our qualitative assessments in Chile, Vietnam, South Africa, and Thailand identified this as an important factor in typhoid fever control. In most countries, unrestricted over-the-counter access to antimicrobial agents was widespread. We do not believe that broad access to antimicrobial use and health system interventions played a role in the reduction of typhoid fever, although widespread availability of antimicrobials such as ciprofloxacin played a role in reducing the expected adverse outcomes from prevalent multiple-drug resistant *Salmonella* Typhi.

An important finding is that despite some reduction in burden in a few settings compared with the 1990s, typhoid fever remains an important disease in many countries, especially in Bangladesh, India, and Pakistan in South Asia, and Nigeria. In some countries typhoid has not yet been eradicated, and there are periodical typhoid fever outbreaks related to travel and migration. Some of the persistent typhoid fever in South Asia may be related to emerging drug-resistant strains of *Salmonella* Typhi, although direct evidence of these having a role in the maintenance of *Salmonella* Typhi in these countries is lacking. No information on human carriage or carriers was assessed in this review, but more knowledge in this area is required to understand and limit the risks of human transmission. An important area to explore with respect to carriage is the prevalence of gallstones as this has been noted as a potential risk factor for developing chronic carriage.^34^

### Implications of findings.

The important finding from our country case studies is that with gains in economic conditions, education, and environmental health, there may be an associated reduction in typhoid fever burden. However, over the last 10–15 years, the effect seems largely notable in countries with higher baseline levels of economic development, female literacy, and investments in public sanitation. In other countries of South Asia and Africa, the reduction of typhoid fever has slowed or the trend is in the opposite direction. We found no clear relationship between growing prevalence of antimicrobial resistance and typhoid fever burden, nor evidence that recent outbreaks are driving overall rates of typhoid fever. In countries where subnational data are available, such as Thailand and South Africa, there is notable diversity of disease occurrence, and rates of reduction of typhoid are evident. It was not possible to determine from this study whether the advent and growth of urban slums played a role in the reduction of typhoid in the case countries with subnational data.

By reviewing national data, there is no single strategy for typhoid control and management. On the other hand, at local levels, the main modes of transmission of typhoid may be better discerned if appropriate epidemiologic investigations and surveillance are undertaken. Countries with high burdens of typhoid must continue to invest in strategies at the local level to address environmental factors, such as 24/7 access to safe drinking water and improved public sanitation, including systems for wastewater treatment that are known to interrupt transmission or diminish the risk of acquiring typhoid. These efforts must also be coupled with strategies to promote food safety, improved access to healthcare services, and address social determinants of health such as education, socioeconomic status, access to safe water, and sanitation.

### Facilities.

Surveillance systems are needed to monitor typhoid trends and characterize the effect of interventions; they are also useful for identifying outbreaks and tracking the spread of antimicrobial resistance. Surveillance systems require investments in laboratory services and appropriate diagnostics, and policies for case management and contact tracing. Lack of appropriate diagnostics such as blood or bone marrow cultures are associated with overuse and prescriptions of antibiotics on clinical suspicion, and could potentially contribute to the observed emergence and increase in drug resistance in *Salmonella* Typhi and other pathogens. We would strongly recommend creating a global repository of information related to typhoid burden and patterns of antimicrobial resistance, which can be based on a network of such surveillance systems in endemic countries.

A clear potential, therefore, exists for appropriate vaccination strategies in endemic countries, especially those with high residual levels of endemic typhoid fever. In countries with low levels of typhoid fever and pockets of disease, vaccination strategies that target populations with the greatest risk may be needed. Existing vaccines, both oral typhoid vaccine (Ty21a) and Vi-capsular polysaccharide vaccine, can be used in school-age children and adults, including travelers. Between 2015 and 2016, a trial was conducted in the United Kingdom with the new Typbar-typhoid conjugate vaccine, the first conjugate vaccine for typhoid fever prevention.^32^ Although not yet licensed, the trial showed promising data as it can be used in children under the age of two.^35^ The results of the trial indicated that the Typbar-TCV had 55% protective efficacy against typhoid fever. With the development of more effective protein conjugate polysaccharide vaccines for younger children, there is substantial potential for longer lasting prevention in younger age groups, potentially as part of the Expanded Program on Immunization schedule in areas with endemic disease where the incidence rate can be high in toddlers and preschool children.

Finally, our review also points out the need for appropriate research to fill many of the knowledge gaps in typhoid. We need to know more about the contribution of chronic carriers to overall transmission in endemic countries, the role of food versus water as vehicles of transmission, and the utility of environmental testing; these important gaps can be addressed in targeted research. Better and more robust epidemiological methods, including molecular genomics as a means of identifying the sources of *Salmonella* Typhi and improved disease surveillance may strengthen our understanding of disease transmission and risks. We have many of the technologies and tools that can help control and dramatically reduce typhoid fever as a major public health problem by 2030, setting us on a path toward its eventual elimination or eradication, if these interventions are widely introduced. Continued work to demonstrate evidence-based, cost-effective strategies for typhoid control will help mobilize the political will and financial resources necessary to achieve the goal of vanquishing this disorder within our lifetimes.
